# Kola nut from *Cola nitida* vent. Schott administered to pregnant rats induces histological alterations in pups’ cerebellum

**DOI:** 10.1371/journal.pone.0247573

**Published:** 2021-03-08

**Authors:** Foluso A. Atiba, Amos A. Fatokun, Innocent O. Imosemi, Adefolarin O. Malomo

**Affiliations:** 1 Department of Anatomy, College of Medicine, University of Ibadan, Ibadan, Nigeria; 2 School of Pharmacy and Biomolecular Sciences, Liverpool John Moores University, Liverpool, United Kingdom; 3 Department of Surgery, College of Medicine, University of Ibadan, Ibadan, Nigeria; Tokyo Medical and Dental University, JAPAN

## Abstract

Kola nut (from *Cola nitida*) is popular in Nigeria and West Africa and is commonly consumed by pregnant women during the first trimester to alleviate morning sickness and dizziness. There is, however, a dearth of information on its effects on the developing brain. This study, therefore, investigated the potential effects of kola nut on the structure of the developing neonatal and juvenile cerebellum in the rat. Pregnant Wistar rats were administered water (as control) or crude (aqueous) kola nut extract at 400, 600, and 800 mg/kg body weight orally, from pregnancy to day 21 after birth. On postnatal days 1, 7, 14, 21 and 28, the pups were weighed, anaesthetised, sacrificed and perfused with neutral buffered formalin. Their brains were dissected out, weighed and the cerebellum preserved in 10% buffered formalin. Paraffin sections of the cerebellum were stained with haematoxylin and eosin for cerebellar cytoarchitecture, cresyl violet stain for Purkinje cell count, Glial Fibrillary Acidic Protein (GFAP) immunohistochemistry (IHC) for estimation of gliosis, and B-cell lymphoma 2 (Bcl-2) IHC for apoptosis induction. The kola nut-treated rats exhibited initial reduction in body and brain weights, persistent external granular layer, increased molecular layer thickness, and loss of Bergmann glia. Their Purkinje cells showed reduction in density, loss of dendrites and multiple layering, and their white matter showed neurodegeneration (spongiosis) and GFAP and Bcl-2 over-expression, with evidence of reactive astrogliosis. This study, therefore, demonstrates that kola nut, administered repeatedly at certain doses to pregnant dams, could disrupt normal postnatal cerebellar development in their pups. The findings suggest potential deleterious effects of excessive kola nut consumption on human brain and thus warrant further studies to understand the wider implications for human brain development.

The developing brain, especially the cerebellum, is highly susceptible to the effects of many types of chemicals and environmental pollutants [1, 2]. The brain is embryologically divided into 3 major parts rostrocaudally: the forebrain (prosencephalon), midbrain (mesencephalon) and hindbrain (rhombencephalon) [3]. The cerebellum develops from the hindbrain, which subdivides into the medulla oblongata caudally (myelencephalon) and the pons and the cerebellum rostrally (metencephalon) [4, 5]. The cerebellum, one of the earliest structures of the brain to differentiate, attains maturity months postnatal [6, 7]. For this reason, the cerebellum is highly susceptible to developmental abnormalities and deviation from normalcy [7, 8]. In rats, some neuroactive chemicals have been shown to induce long-lasting behavioural and structural alterations to the brain, even at low doses, especially at critical developmental periods [9–11]. Therefore, the study of the potential neurodevelopmental effects of chemicals which organisms, including humans, are commonly exposed to, either through environmental contact, direct ingestion from food substances or some other means, is extremely important. Medicinal plants or their products are a major source of chemicals with potential neurodevelopmental effects. One of such plant is the kola tree.

The kola tree (*Cola nitida*) is known to West Africa, especially Sierra Leone, Liberia, Ivory Coast and Nigeria. Its fruit is kola nut, called “Obi” by the Yoruba tribe of Nigeria [12, 13]. Some of the chemical constituents of African kola nut include caffeine, theobromine, d-catechin, l-epicatechin, kolatin, kolanin, glucose, starch, fatty matter, tannins, anthocyanin pigment, betaine and protein [14, 15]. Kola nut has been used as aphrodisiac and appetite suppressant [16], as it has been found to cause decrease in food and water intake [17]. Other studies have investigated its effects on the heart, liver and kidney [18], stomach [19, 20], male reproductive system [21, 22], behavioural and endocrine system [13, 23–26], body weight [13, 17], the brain [27], and brain sodium pump activity [28]. Kola nut is also one of the herbal fruits that get consumed by pregnant women in Nigeria [29, 30]. However, despite these various studies done on kola nut, there is a dearth of information on its effects on the developing brain.

About 82.8% of pregnant women experience a phenomenon known as morning sickness during pregnancy [31]. Morning sickness usually comprises nausea, vomiting, tiredness and spitting, and it is more intense in the morning. For some women, it occurs only during the first trimester, while for some others it might occur throughout the gestational period. Studies have shown that a good percentage of these women resort to taking any substance that can reduce the effects of morning sickness [32]. Amongst the substances consumed in pregnancy is kola nut [29, 30], although why this is so has not been fully documented by any study. However, based on selected interviews, some pregnant women confessed to taking kola nut to suppress early morning sickness symptoms. It is of note that the brain development of the foetus is critical during the first trimester, as this is when the neural tube folds, elongates, expands and undergoes cytological changes to attain the typical brain arrangement of the cerebrum, cerebellum and the brain stem. This study was, therefore, carried out to investigate the potential effects of kola nut on the developing brain, with a focus on the cerebellum. It was found that *in utero* exposure of foetus to repeated administration of certain doses of kola nut could induce abnormal changes in the neonatal cerebellum. While we found the effects of kola nut to be widespread in the brain, we specifically investigated the cerebellum, as it is an important model for brain development and evolution and has a key role in sensory-motor processing, thus providing us an opportunity to demonstrate a proof of concept.

## Materials and methods

### Ethical approval

Ethical approval for this project was obtained from the Institutional Animal Care and Use Committee, University of Ibadan, Nigeria (UI-ACUREC/APP/2015/009), and the use of animals in the study was consistent with internationally accepted standards for the welfare of, and experimentation on, animals (e.g., NIH Guide for the care and use of laboratory animals), as described in the methodology.

### Collection and identification of plant material

Fresh kola nut (*Cola nitida*) fruits were purchased from kola plantation in Sagamu, Ogun State, Nigeria in August 2016. The fruits of kola nut were identified and authenticated at the Federal Research Institute of Nigeria (FRIN), with Federal Herbarium Identification (FHI) Number 109605.

### Preparation of kola nut extract

The kola nut fruits were cut into small cubes and room dried until a constant weight was achieved. These were ground with a miller and mixed with water, with 0.5% of chloroform added to avoid the growth of fungi. The soaked kola nut was vortexed every one hour for 12 hours. This was sieved and filtered with Whatman filter paper 4. The supernatant was concentrated using a rotary evaporator (which should remove the chloroform) and the concentrate was freeze-dried and kept in the refrigerator at +4°C, which ensured the stability of its composition. The kola nut extract was reconstituted with water and administered to the pregnant rats every morning through oral gavage, in doses of 400, 600 and 800 mg/kg body weights as previously reported [1]. Administration through oral gavage ensured the entire extract was directly delivered into the stomach of the dams. The dams were then monitored for any signs of vomit or regurgitation (there was none) before leaving them in their respective cages.

### Experimental animals

Sexually-matured female and male Wistar rats were purchased from the central Animal House of the Faculty of Veterinary Medicine, University of Ibadan, Ibadan, Nigeria. The animals were acclimatized for two weeks in the central Animal House under 12-hour night and day cycles and fed with rodents’ cubes purchased from Ladokun Feeds, Ibadan, Oyo State, Nigeria, and water was provided *ad libitum*.

Forty (40) sexually matured female Wistar rats weighing between 160 g-180 g were designated for the study. The female rats were mated by matured male rats and pregnancy was noted by presence of vaginal plug and confirmed by vaginal smear. However, not all the dams carried their pregnancy to term; therefore, seven (7) dams were used in each of the four experimental groups (see following section). The number of animals used was what was required in order to ascertain statistical significance of any effect.

### Grouping of animals for experiments

The animals were randomly grouped into four groups (Groups 1–4) of seven rats each:

#### Group 1

Pregnant animals received 0.4 ml of distilled water orally as placebo, from embryonic day (ED) 1 of pregnancy to post-natal day (PND) 21, to serve as Control (CON).

#### Group 2 (Treated (TRT)) 1

Pregnant animals received 400 mg/kg body weight of kola nut extract orally from ED1 of pregnancy to PND 21.

#### Group 3 (TRT2)

Pregnant animals received 600 mg/kg body weight of kola nut extract orally from ED1 of pregnancy to PND 21.

#### Group 4 (TRT3)

Pregnant animals received 800 mg/kg body weight of kola nut extract orally from ED1 of pregnancy to PND 21.

The pregnant animals were kept under close observation before and during pregnancy and after delivery, and animal use was in accordance with local and national guidelines, consistent with the United States National Institutes of Health (NIH) Guidelines for the Care and Use of Laboratory Animals in Biomedical Research [33].

### Sacrifice of animals and tissue processing for histological studies

Five pups of post-natal days 1, 7, 14, 21 and 28 from each group were weighed, anaesthetized by intraperitoneal administration of 300 mg/kg ketamine (Pfizer, USA) and 5 mg/kg diazepam (Pfizer, USA), perfused with neutral buffered formalin (NBF), and their brains dissected out and weighed. The brains were preserved in Bouin’s fluid (75 ml of saturated picric acid, 25 ml of formalin and 5 ml of acetic acid) for 6 h and then transferred to 10% buffered formaline for ease of grossing and to prevent autolysis by enzymatic or bacterial actions; another set of five pups of post-natal days 1, 7, 14, 21 and 28 from each group were also weighed, anaesthetized and perfused with NBF, their brains dissected out, weighed and preserved in NBF to preserve their structure and molecular composition.

A mid-sagittal section of the cerebellum was dissected. The sections were dehydrated through ascending grades of alcohol, 70%, 90%, 95% and two changes of absolute alcohol for 1 hour each. Clearing of the tissues was done in two changes of xylene for thirty minutes each. The sections were then infiltrated in four changes of molten paraffin wax for an hour each at a temperature of 60°C. Immediately after the infiltration stage, the tissues were embedded in molten paraffin wax with embedding moulds and allowed to solidify into blocks ready for sectioning.

Sections of 5 micrometers (5 μm) were cut using a rotary microtome. The cut sections were floated in a warm water bath at a temperature of 30–40°C and picked up on slides coated with egg albumin, which acted as a mountant allowing the cut sections to stay for Haematoxylin and Eosin (H&E), while Poly-L-lysine slides were used for Cresyl violet and Immunohistochemistry staining. The slides were dried on a regulated hot plate (57°C).

### Haematoxylin and Eosin (H&E) staining

This was performed (as well as cresyl violet staining and immunohistochemistry (IHC)) at the Histopathology Laboratory, National Hospital, Abuja, Nigeria (laboratory contact: Dr Jonathan Madueke). Sections were placed in xylene to dissolve the paraffin wax. They were then washed in absolute alcohol for a minute to remove xylene and hydrated in two changes of descending grades of alcohol (90%, 70%) and distilled water for five minutes each, after which they were washed in running tap water and stained with Haematoxylin for fifteen minutes. Sections were then differentiated in 1% acid alcohol for thirty seconds, placed in Scott’s tap water for 1 min for bluing, counterstained with eosin for a minute, rinsed briefly in tap water and transferred to ascending grades of alcohol (70%, 95%) for two minutes each and into two changes of absolute alcohol for a minute each. They were cleared in xylene and mounted in Dibutylphthalate Polystyrene Xylene (DPX) with cover slips in their wet state, at what stage they were ready for microscopic examination.

### Cresyl violet staining

The serial sections of the cerebellum were mounted on Poly-L-lysine-coated adhesive glass slides and allowed to dry over a slide warmer. The sections were transferred to a slide rack and put in a chloroform/ethanol (4:1) solution for 1 h and then placed in cresyl violet acetate solution (Sigma-Aldrich, Germany) for 10 min, with careful monitoring of the colour of the tissue, to avoid overstaining. They were dehydrated by passing through ascending concentrations of ethanol, 50%, 70%, 80%, 90%, 96% and 100% twice for 1 minute each. The sections were then passed through two changes of xylene for 10 minutes each, cover-slipped with DPX Mountant (BDH Chemicals Ltd., England) and allowed to dry.

### Immunohistochemistry (IHC)

Immunohistochemistry (IHC) using the primary antibodies anti-GFAP (mouse monoclonal, clone G-A-5, Bio SB, CA, USA) and anti-Bcl-2 (mouse monoclonal, clone Bcl2-100, EnVision, DAKO (now Agilent), Germany) was done on paraffin-embedded tissue. Immunohistochemical detection was done using the Avidin-Biotin Complex (ABC) method (Avidin-Biotin Immunoperoxidase method). The Ultra-Sensitive ABC Peroxidase Rabbit IgG Staining Kit used (Cat. No. 32054, Lot UG282613; Thermo Scientific, MA, USA) came with 3,3′-Diaminobenzidine (DAB), haematoxylin and biotinylated secondary antibodies. The antibody dilution factor used was 1:100 for all the markers.

The processed tissue was sectioned at 5 micrometers (5 μm) on the rotary microtome, mounted on Poly-L-lysine slides, and placed on the hot plate at 70°C for at least 1 h. Sections were dewaxed and hydrated. Antigen retrieval was performed on the sections by heating them for 15 min in a citric acid solution of pH 6.0 using a microwave at a power of 100 W and they were equilibrated gradually with cool water to displace the hot citric acid for at least 5 min for the section to cool. Peroxidase blocking was done on the sections by covering them with 3% hydrogen peroxide (H_2_O_2_) for 15 min and they were washed with phosphate-buffered saline (PBS) and protein blocking was performed using avidin for 15 min. Sections were washed with PBS and endogenous biotin in tissue was blocked using biotin for 15 min. After washing with PBS, sections were incubated with the respective diluted primary antibody (diluted 1:100) for 60 min and excess antibody was washed off with PBS and a secondary antibody (LINK) was applied on the section for 15 min. Sections were washed and the Label, horseradish peroxidase (HRP), was applied on them for 15 min. A working 3,3′-Diaminobenzidine (DAB) solution, made up by mixing 1 drop (20 μl) of the DAB chromogen with 1 ml of the DAB substrate, was applied on the sections (for a positive target a brown reaction was expected to begin to appear at this moment) after washing off the HRP with PBS for at least 5 min. Excess DAB solution and precipitate were washed off with water. Sections were counterstained with Haematoxylin solution for at least 2 min and blued briefly (1 min) by immersion in Scott’s tap water until the stain was intensified in the target nuclei. They were then dehydrated in alcohol, cleared in xylene and mounted in DPX. Cells with specific brown colours in the cytoplasm, cell membrane or nuclei (depending on the antigenic sites) were considered to be positive, while the haematoxylin-stained cells without any form of brown colours were scored negative. Non-specific binding/brown artifacts on cells and connective tissue were disregarded. Quantification of the intensity of antibody signalling was done using ImageJ software.

### Measurement of gross and histomorphometric parameters

Body weight and brain weight of the pups were measured using a Mettler Analytical Balance. Morphological changes within the cerebellar cortex were assessed on a 500 pixel Leica microscope. For histomorphometry, the Motic software 2.0 was used to determine molecular layer thickness in μm and the purkinje density in cells/μm^2^ (total number of purkinje cells in a given area).

### Data presentation and statistical analyses

The brain sections processed for Haematoxylin & Eosin (H & E), Cresyl and immunohistochemistry were viewed under Leica application suit version 3.3.0 (Switzerland) X 40 objective. All values of parameters were expressed as mean ± SEM (standard error of mean) and subjected to statistical analysis (through GraphPad Prism 6.0) employing one-way ANOVA, followed by Tukey post-hoc test at 95% confidence interval. Statistical significance was established at P<0.05. Levels of significance are depicted as *P<0.05, **P<0.01, ***P<0.001 and ****P<0.0001 for the indicated comparisons, where CON = Control, and each TRT indicated the administered dose of kola nut, as follows: TRT1 = 400 mg/kg body weight; TRT2 = 600 mg/kg body weight; and TRT3 = 800 mg/kg body weight.

## Results

### Behavioural and weight changes in kola nut-administered pregnant dams

It was observed that pregnant dams administered the kola nut extract were more active and aggressive than the negative control group dams that did not receive the extract. There was also evidence of impairment of motor control and posture (balancing) in kola nut-treated dams, as they showed some difficulty in walking on the grid and flared their hind limbs when picked by the tails. Kola nut administration was for a total of 6 weeks, starting at mating (beginning of pregnancy), continuing through the 3 weeks of pregnancy, and then ending 3 weeks after the pups were born (post-natal day (PND) 21), which was when the pups were weaned. Some days after the commencement of daily administration of the kola nut extract, we noticed some reduction in water and food intake of kola nut-treated pregnant dams. Then, the same week the dams had given birth (3 weeks after kolanut administration commenced), we began to notice weight loss in the kola nut-administered dams. All dams had similar weights before and during pregnancy (between 170–175 g at the time of giving birth). The weight loss in kola nut-administered dams that began the week they gave birth was such that, two weeks after giving birth, the average weight (Mean ± SEM) of the negative control dams was 203.6 ± 5.7 g, while the average weights of dams administered 400, 600 and 800 mg/kg kolanut were 163.7 ± 4.1 g, 153.0 ± 2.7 g and 148.6 ± 2.7 g, respectively.

### Gross morphometry: Body weight

**Fig 1:**
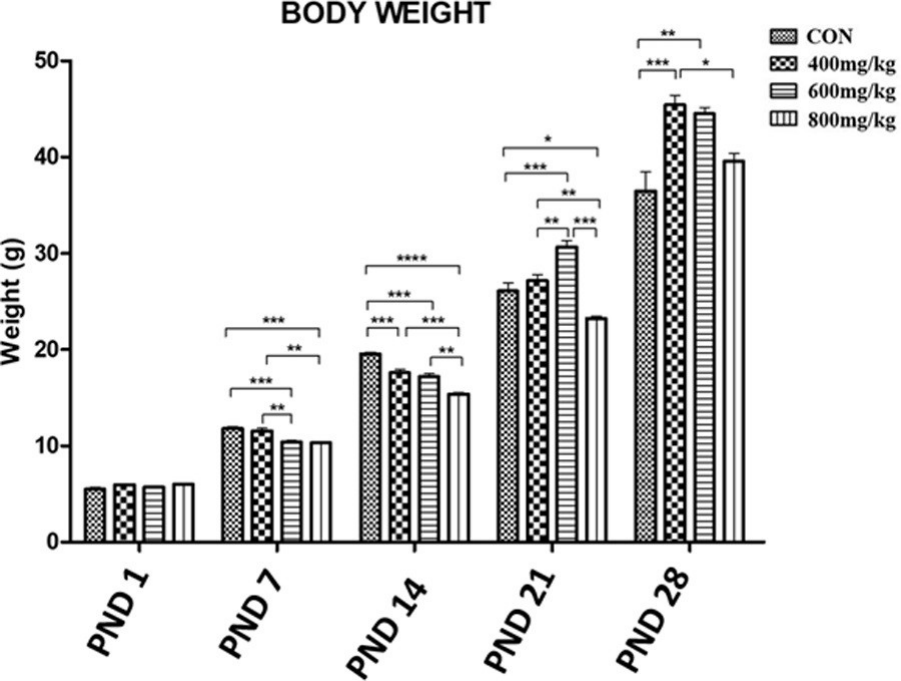
Mean body weights of the control group and the kola nut-treated groups at PND 1, 7, 14, 21 and 28 for n = 5 pups. *P<0.05, **P<0.01, ***P<0.001 and ****P<0.0001 for the indicated comparison.

The mean body weight (g) of the control pups was significantly higher than those of the kola nut-treated (TRT1, TRT2, TRT3) pups on PND7 (control: 11.78 ± 0.19, vs. 11.58 ± 0.27, 10.40 ± 0.15 and 10.32 ± 0.16, respectively) and PND14 (control: 19.56 ± 0.19, vs. 17.60 ± 0.35, 17.18 ± 0.37 and 15.38 ± 0.18, respectively), but lower than that of 600 mg/kg kola nut treatment on PND21 (control: 26.10 ± 0.85, vs. 30.66 ± 0.70) and 400 and 600 mg/kg kola nut treatments on PND28 (control: 36.48 ± 2.00, vs. 45.42 ± 1.01 and 44.50 ± 0.66, respectively) (Fig 1).

### Gross morphometry: Brain weight

**Fig 2:**
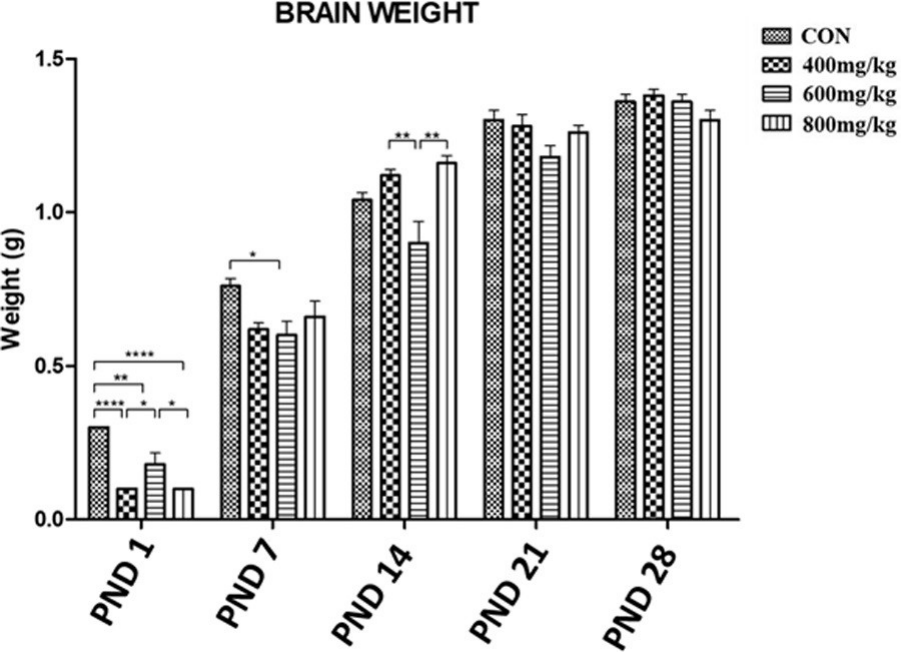
Mean brain weights of the control group pups and the kola nut-treated group pups at PND 1, 7, 14, 21 and 28 for n = 5 pups. *P<0.05, **P<0.01 and ****P<0.0001 for the indicated comparison.

The mean brain weight (g) of control pups was significantly higher than those of kola nut-treated (TRT1, TRT2, TRT3) pups on PND1 (control: 0.30 ± 0.00, vs. 0.10 ± 0.00, 0.18 ± 0.03 and 0.10 ± 0.00, respectively) and PND7 (control: 0.76 ± 0.02, vs. 0.62 ± 0.02, 0.60 ± 0.04 and 0.66 ± 0.05, respectively), while on PND14 the mean brain weights (g) of kola nut-treated pups at 400 and 800 mg/kg (1.12 ± 0.02 and 1.16 ± 0.02, respectively) were higher than that of the control pups (1.04 ± 0.02). However, there was no significant difference between the mean brain weights of control and kola nut-treated pups on PND21 (control: 1.30 ± 0.03) and PND28 (control: 1.36 ± 0.02) (Fig 2).

### Histomorphology

**Fig 3:**
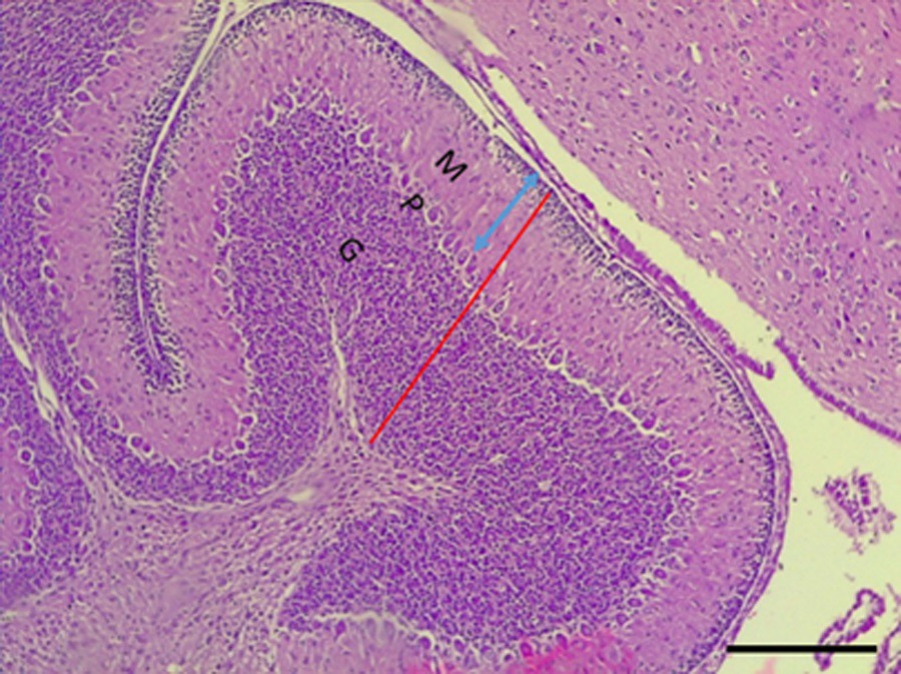
Demarcations of layers of the cerebellum of control and kola nut extract-treated pups. A microphotograph of the mid-sagittal section of a hematoxylin and eosin-stained folium surface of the cerebellar cortex from a control (PND14) rat pup. The red line shows the cortical thickness of the folium surface comprising the molecular (M), purkinje (P) and granular (G) layers, while the blue double arrow shows the molecular layer (M) thickness. Scale bar = 40 μm.

Measurements were made consistently in the anterior lobe of the cerebellum. The demarcations of the molecular, purkinje and granular layers and how the thickness of the molecular layer was measured are shown in Fig 3.

**Fig 4:**
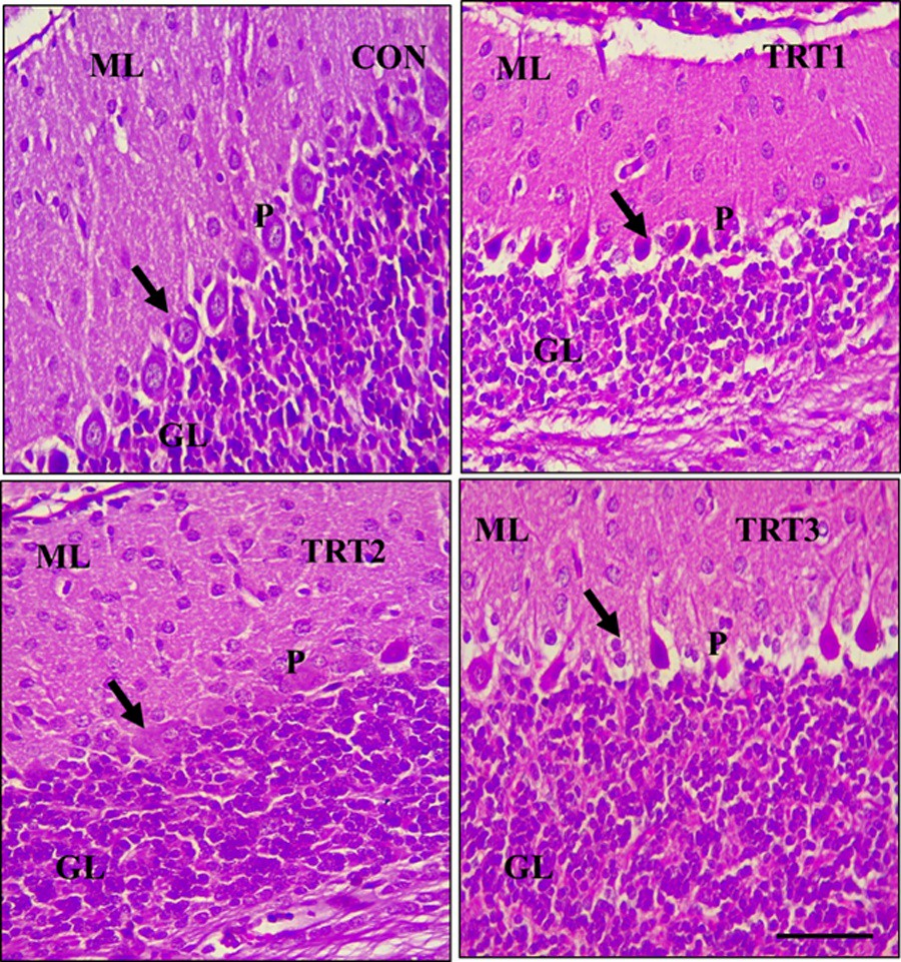
Mid-sagittal sections of formalin-fixed, paraffin-embedded, H&E stained cerebellum of control (CON) and kolanut extract-treated (TRT) PND 21 pups. The three layers of the cerebellar cortex are shown (ML is Molecular Layer; P is Purkinje; GL is Granular Layer). CON shows a normal cytoarchitecture of the cerebellum with viable Purkinje cells *(black arrow)*. TRT1 and TRT3 show a Purkinje cell layer that is comprised of pyknotic and degenerated Purkinje cells (*black arrow*) and TRT2 shows a sparsely populated Purkinje cell layer *(black arrow)*. Scale bar = 40 μm.

Treatments with 400 mg/kg, 600 mg/kg and 800 mg/kg kola nut are depicted as TRT1, TRT2 and TRT3, respectively. Purkinje cells of the kola nut-treated groups on PND21 showed varying degrees of degeneration and necrosis compared to the control group of the same age. The cells were pyknotic, with chromatolysis, while some regions showed total loss of Purkinje cells (Fig 4).

**Fig 5:**
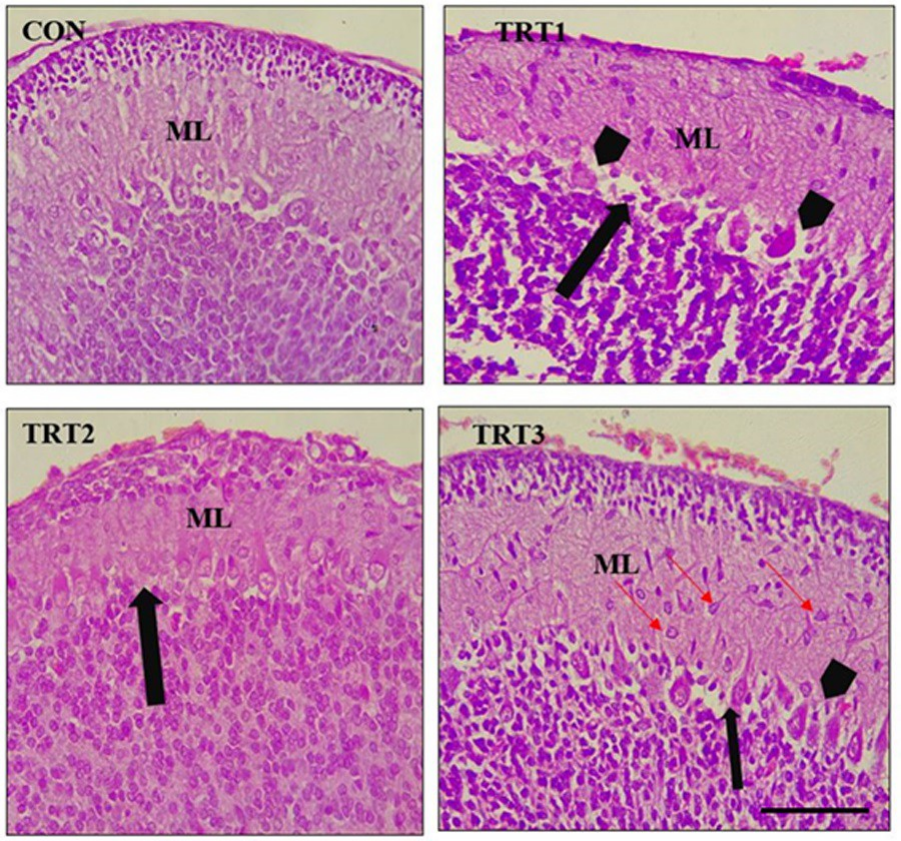
Mid-sagittal sections of formalin-fixed, paraffin-embedded, H&E stained cerebellum of PND 14 pups. CON shows a normal molecular layer (ML) with few cells scattered in the parenchyma. TRT1 shows thinner ML with a mild separation between the ML and granular layer *(black arrow)* with pyknotic and degenerated purkinje cells *(black arrow heads)*. TRT2 shows thinner ML with sparse Purkinje cells *(black arrow)*. TRT3 shows relatively normal ML with various morphologies *(red thin arrow)* in the parenchyma, slight separation of the ML and granular layer *(black arrow)*, and pyknotic purkinje cells with central chromatolysis *(black arrow head)* Scale bar = 40 μm.

The normal architecture of the molecular layer was also disrupted. Cells with different morphologies were seen scattered in the molecular layer of the kola nut-treated groups, especially in the TRT3 on PND 14 (red arrows), with mild separation of the molecular layer (black arrow) (Fig 5).

**Fig 6:**
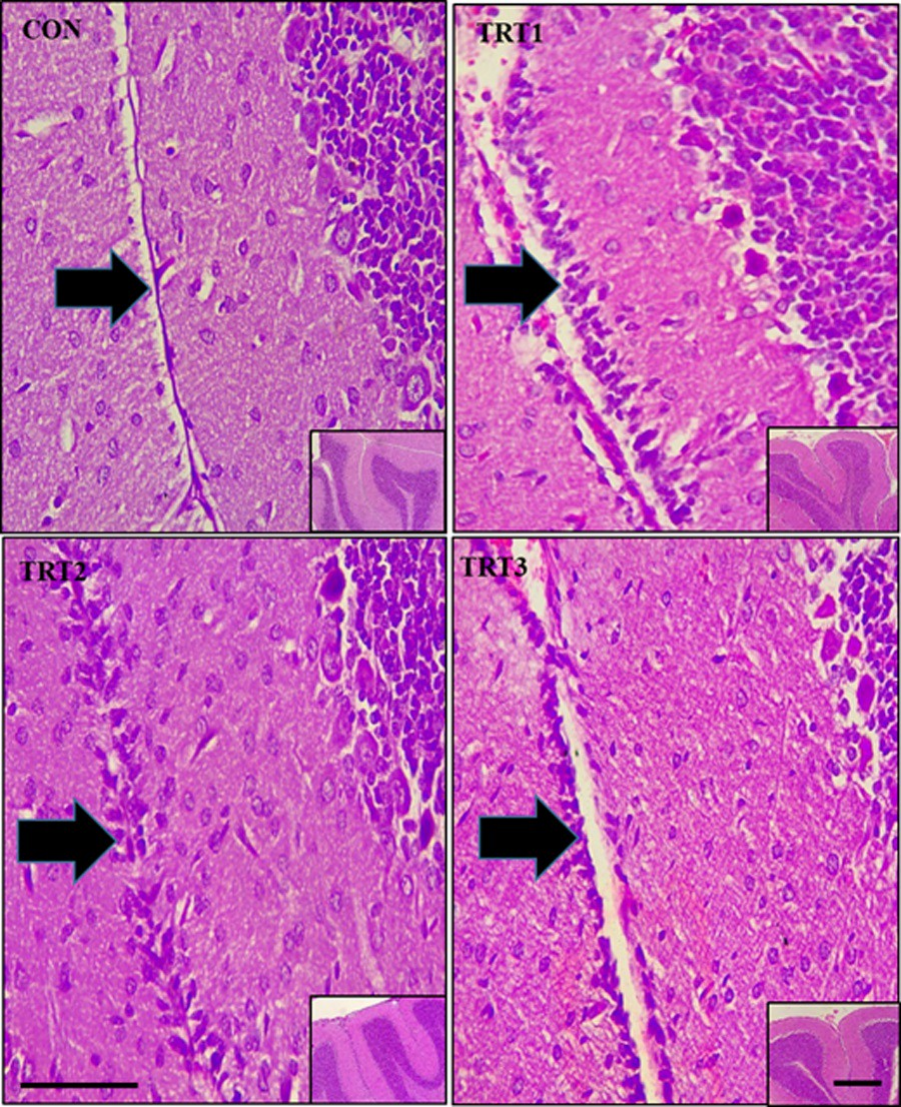
Midsagittal sections of formalin-fixed, paraffin-embedded, H&E stained cerebellum of PND 21 pups. Slide CON shows the absence of external granular layer (EGL) (arrow). Slides TRT1, TRT2 and TRT3 show retention of external granular layer (EGL) (arrow) following the ingestion of kola nut extract. The insets show the lower magnification of the photomicrographs. Scale bar = 40 μm for main image and 60 μm for inset.

Cells from the external granular layer (EGL) migrate to the granular layer at PND 20 in rats and by PND 21 the EGL is expected to have completely migrated. However, the EGL was retained in all the kola nut-treated groups at PND 21 (Fig 6).

**Fig 7:**
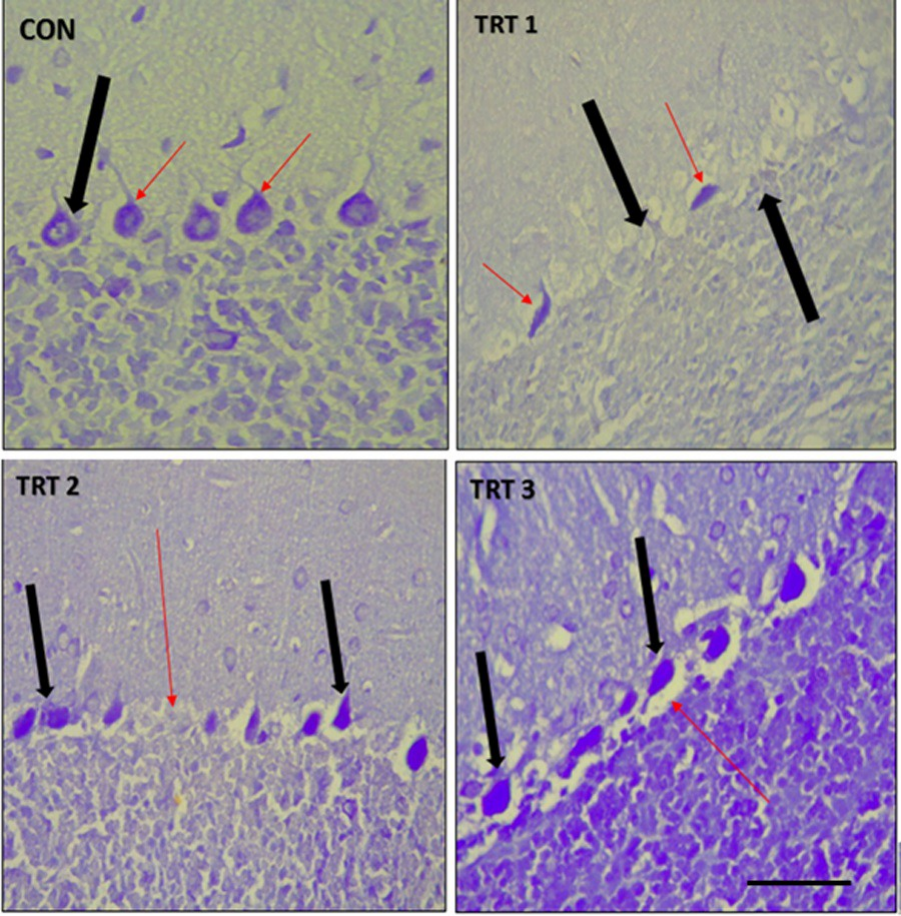
Mid-sagittal sections of formalin-fixed, paraffin-embedded, Nissl-stained cerebellum of PND 28 pups. CON shows a normal cytoarchitecture of the cerebellum with viable Purkinje cells and normal flask-shaped morphology (black arrow). TRT1 shows loss of Purkinje cells and pyknotic cells (red arrow). TRT2 and TRT3 show Purkinje cell layer with pyknotic and degenerated cells and loss of normal morphology (red arrow). Scale bar = 40 μm.

In the control group, the Nissl stain showed normal cytoarchitecture of the cerebellum, with viable Purkinje cells (*black arrow*) and normal flask-shaped morphology *(red arrow)*. In contrast, TRT1 showed loss of Purkinje cells (*black arrow)* with pyknotic cells *(red arrow)* and TRT2 and TRT3 showed a Purkinje cell layer comprised of rounded, pyknotic and degenerated cells (*black arrow*) (Fig 7). Red arrow shows loss of Purkinje cells in TRT2 and separation of the molecular and granular layers in TRT3 (Fig 7).

**Fig 8:**
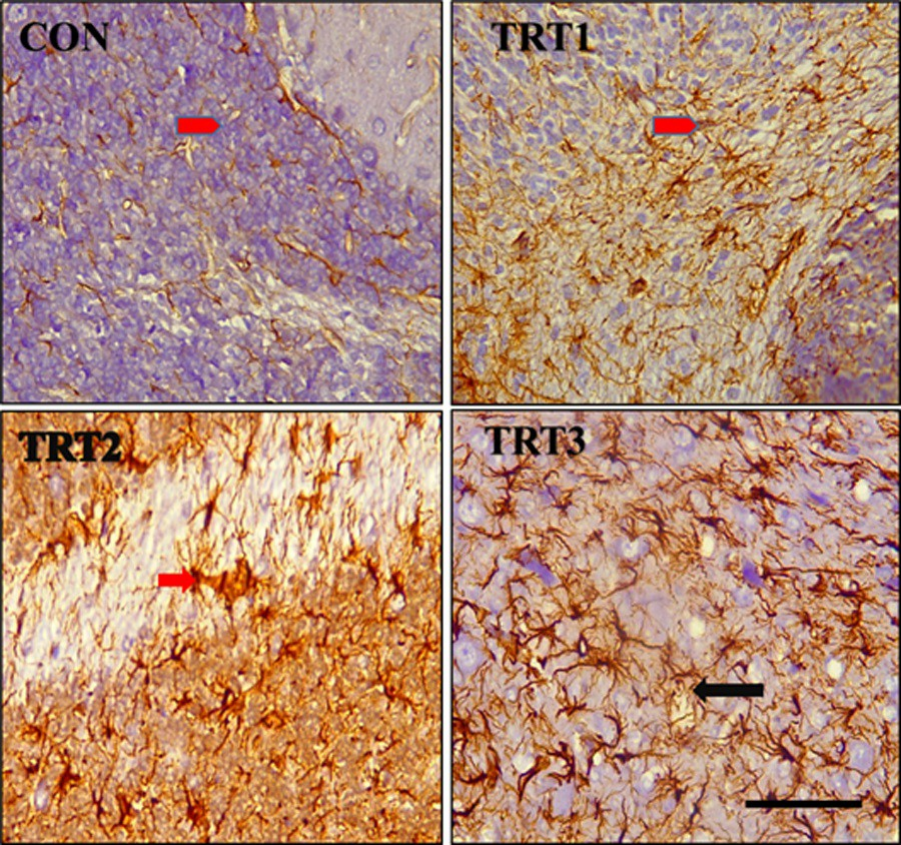
Midsagittal sections of the cerebellar cortex of PND 21 pups immunostained with anti-GFAP antibody. CON shows astrocytes, most of which are not expressing detectable levels of GFAP (*red arrowhead*) within the granular layer. TRT1 shows upregulation of GFAP with astrocyte hypertrophy (*red arrow head*) and mild overlap of domain, while TRT2 and TRT3 show severe diffuse reactive astrogliosis with pronounced upregulation of GFAP, disruption of individual astrocyte domain, astrocytic hypertrophy and proliferation *(red arrow*) as well as pronounced overlap of astrocyte processes (*black arrow*). Scale bar = 40 μm.

**Fig 9:**
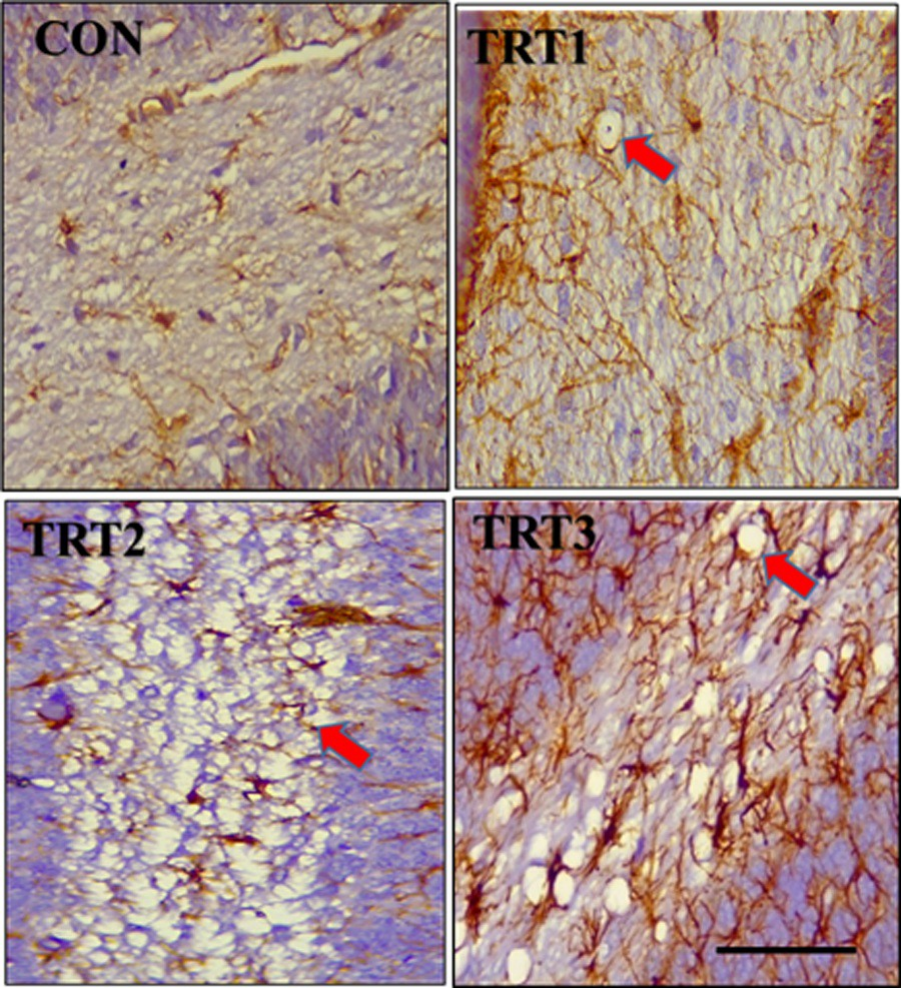
Midsagittal sections of the cerebellum of PND 21 pups stained with anti-Glial Fibrillary Acidic Protein (GFAP) antibody. Slide CON shows absence of spongiosis in the white matter of the cerebellar folium of control pups. Slides TRT1, TRT2 and TRT3 show the presence of mild to severe spongiosis caused by degeneration of white matter following the ingestion of kola nut extract *(red arrow)*. Scale bar = 40 μm.

**Fig 10:**
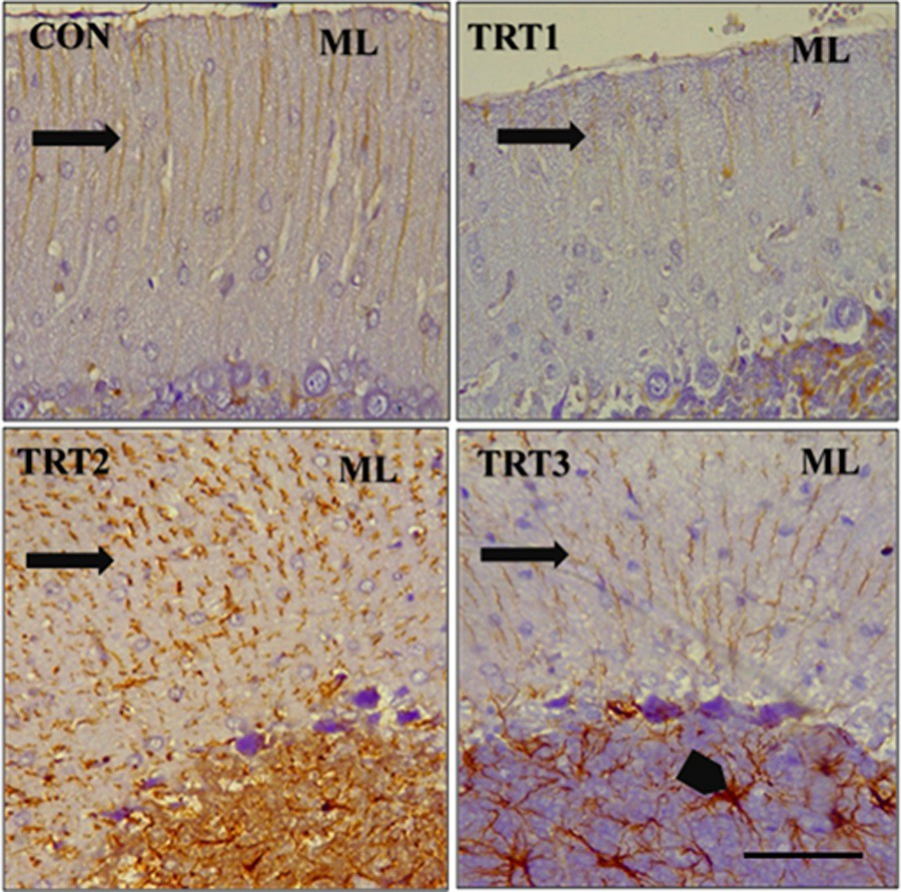
Photomicrographs of the cerebellar cortex of PND 28 pups immunostained with anti-GFAP antibody. CON shows proper layering of the Bergmann glia (*arrow)* in the molecular layer (ML) of the control pups. TRT1 shows a loss of the Bergmann glia depicted by upregulation of GFAP reactivity *(black arrow)*. TRT2 shows an upregulation of GFAP reactivity as well as a severe disruption in the layering of the Bergmann *(black arrow)*. TRT3 shows a mild disruption in the normal layering of the Bergmann glia *(black arrow)*. *Note the hypertrophic astrocyte with overlapping processes (black arrow head)*. Scale bar = 40 μm.

Immunohistochemistry using anti-GFAP (Glial Fibrillary Acidic Protein) antibody on PND 21 cerebellar cortex revealed varying degrees of astrogliosis in the groups that were treated with kola nut extract. This was characterized by upregulation of GFAP reactivity in the astrocytes, hypertrophy, and overlap of astrocyte process with no preservation of individual domains, as well as scar formation (Fig 8). Immunohistochemistry with anti-GFAP antibody also depicted a dose-dependent presence of spongiosis (red arrow) within the white matter of the cerebellum of kola nut-treated PND 21 groups (Fig 9). In addition, immunohistochemistry with anti-GFAP antibody on PND 28 revealed a disruption in the normal layering of the Bergmann glia of treated groups. This disruption was, however, dose-dependent, with pronounced upregulation in the TRT3 group (Fig 10). Note the hypertrophic astrocyte with overlapping processes (black arrow head).

**Fig 11:**
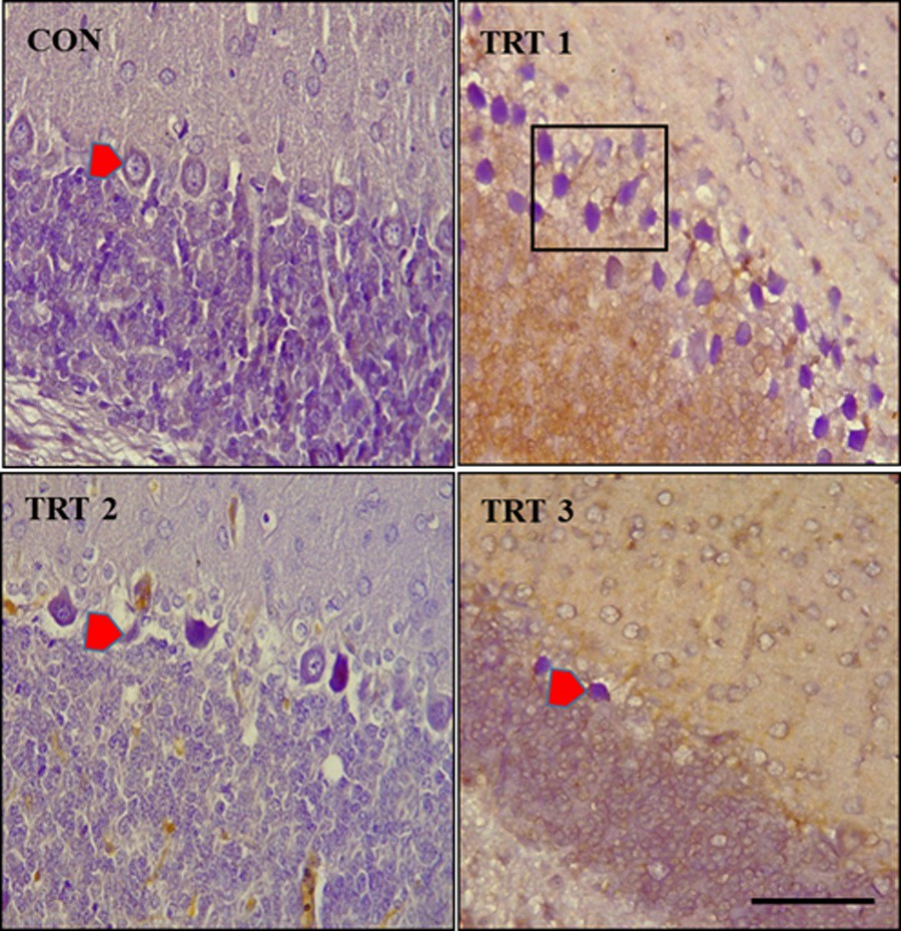
Formalin-fixed, paraffin-embedded cerebellum stained with anti-*Bcl-2* monoclonal antibody. CON shows mild expression of *Bcl-2* and normal architecture of Purkinje cells. TRT1 shows layering of the Purkinje cells (rectangular box) with an overexpression of *Bcl-2* protein in neurones. TRT2 shows necrotizing and necrotized Purkinje cells (*red arrow head*). TRT3 shows necrotized Purkinje cells with high expression of *Bcl-2* protein. Scale bar = 40 μm.

Immunohistochemistry with anti-*Bcl-2* on PND 28 revealed overexpression of the Bcl-2 protein in the kola nut-treated groups and a layering of the Purkinje cells was pronounced in the TRT1 group (Fig 11).

### Histomorphometry

#### Purkinje cell density

**Fig 12:**
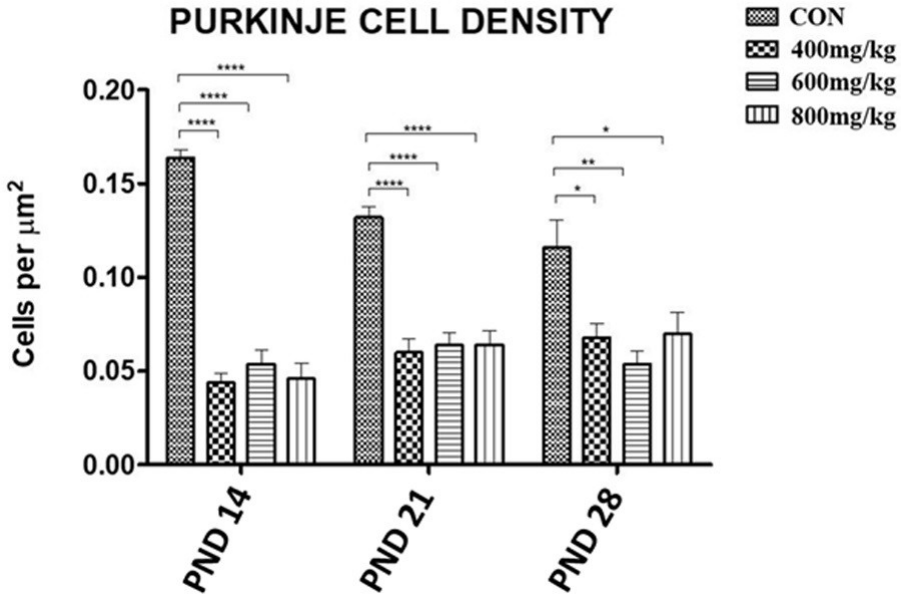
Purkinje cell densities of the cerebella of the control group (CON) and the kola nut-treated groups (400, 600, 800 mg/kg) at PND 14, 21 and 28 (n = 5 pups). *P<0.05, **P<0.01 and ****P<0.0001 for the indicated comparison.

Histomorphometric analysis revealed a progressive decrease in the control (CON) purkinje cell density from PND14 to PND 28. For PND 14, 21 and 28, there was a statistically significant decrease in the purkinje cell density of all the kola nut-treated groups compared to the control. For PND14, the purkinje cell density for the control (CON) was 0.164 ± 0.004 cells/μm2 and there was a significant 73% reduction in the purkinje cell density in the TRT1 group compared to the control (P<0.0001), a value that was not significantly different from the purkinje cell density values obtained for the TRT2 and TRT3 groups. A similar trend was observed for PND21 and PND28, where there was a 55% decrease and a 41% decrease, respectively, in the purkinje cell density for the TRT1 group, and the effect was sustained in the respective TRT2 and TRT3 groups (Fig 12).

#### Molecular layer thickness

**Fig 13:**
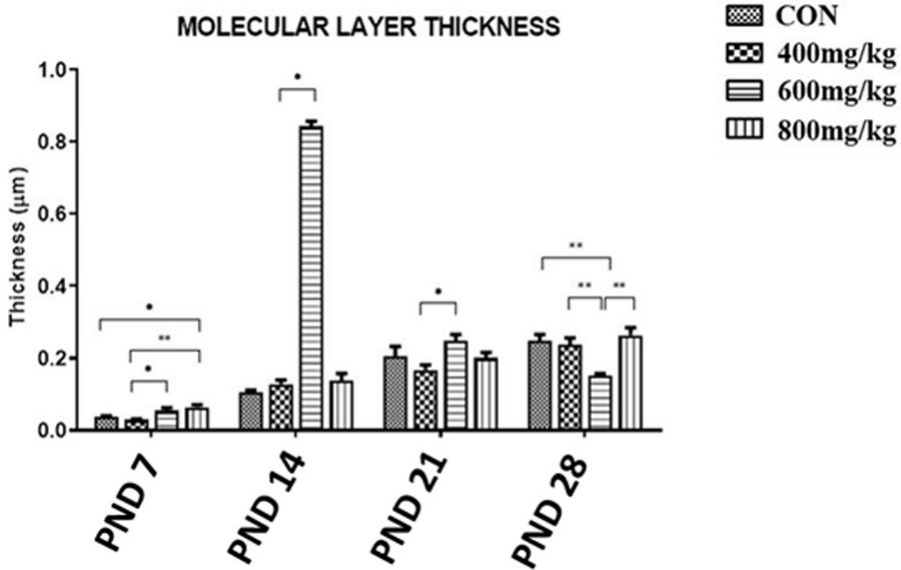
The thickness of the molecular layer of the cerebellum in the control (CON) and kola nut-treated (400, 600, 800 mg/kg) groups at PND 7, 14, 21 and 28 (n = 5 pups). *P<0.05 and **P<0.01 for the indicated comparison.

The molecular layer thickness in the control group (CON) increased steadily from PND7 to PND28, with values of 0.037 ± 0.004μm, 0.106 ± 0.005μm, 0.206 ± 0.027μm and 0.248 ± 0.018μm, respectively. Kola nut extract produced a dose-dependent change in the thickness of the molecular layer. On PND 7, the molecular thickness increased dose-dependently, with the thickness of the TRT3 group being significantly higher than that of the control. Strangely, on PND14, the molecular layer thickness of the TRT2 group was significantly higher than that of the control but that of the TRT1 or TRT2 group was not. The molecular layer thicknesses on PND 21 and 28 were not significantly higher than for their respective controls. On PND 28, the molecular layer thickness of the TRT2 group was significantly lower than that of the control (P<0.01). (Fig 13).

#### GFAP reactivity

**Fig 14:**
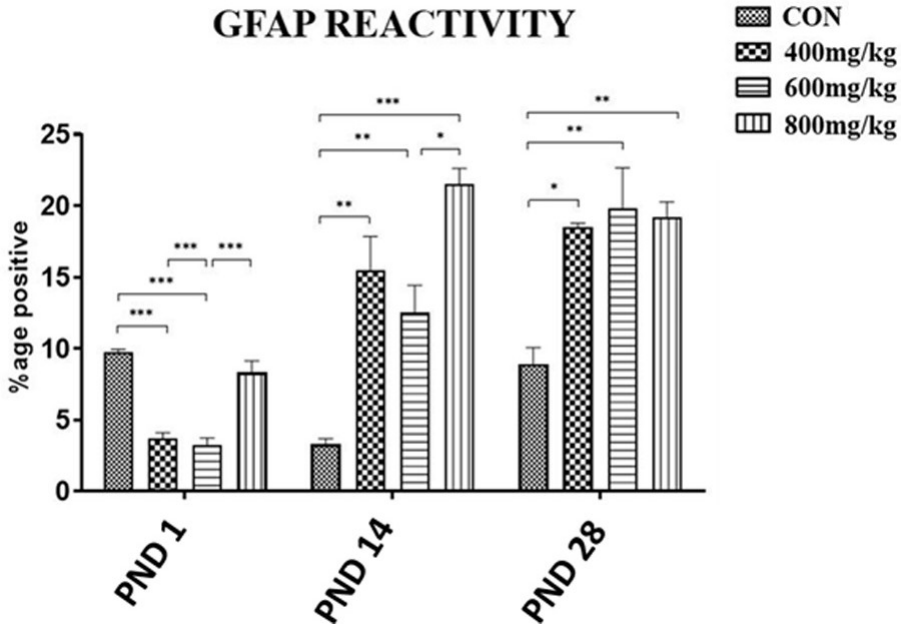
Changes in GFAP expression of both the control (CON) and kola nut-treated (400, 600, 800 mg/kg) groups at PND 1, 14 and 28 (n = 5 pups). *P<0.05, **P<0.01 and ***P<0.001 for the indicated comparison.

GFAP reactivity showed a dose- and age-dependent difference when the kola nut-treated groups were compared to control. Control GFAP reactivity on PND1 was 9.78 ± 0.17% positive. This decreased 3-fold on PND14 but on PND28 was restored to the PND1 level. On PND1, control (CON) GFAP reactivity was significantly higher (nearly three-fold) (P<0.001) than was obtained for the TRT1 or TRT2 group, but was not different from that of the TRT3 group. However, the trend was reversed on PND14 and PND28, as GFAP reactivities in the treatment groups were higher than in the control. On PND14, GFAP reactivities for the TRT1, TRT2 and TRT3 groups were nearly five-, four- and seven-fold higher than in the control (P<0.01, P<0.01 and P<0.001, respectively), while on PND28 they were each 2-fold higher (P<0.05, P<0.01, P<0.01, respectively) (Fig 14).

#### Bcl-2 protein expression

**Fig 15:**
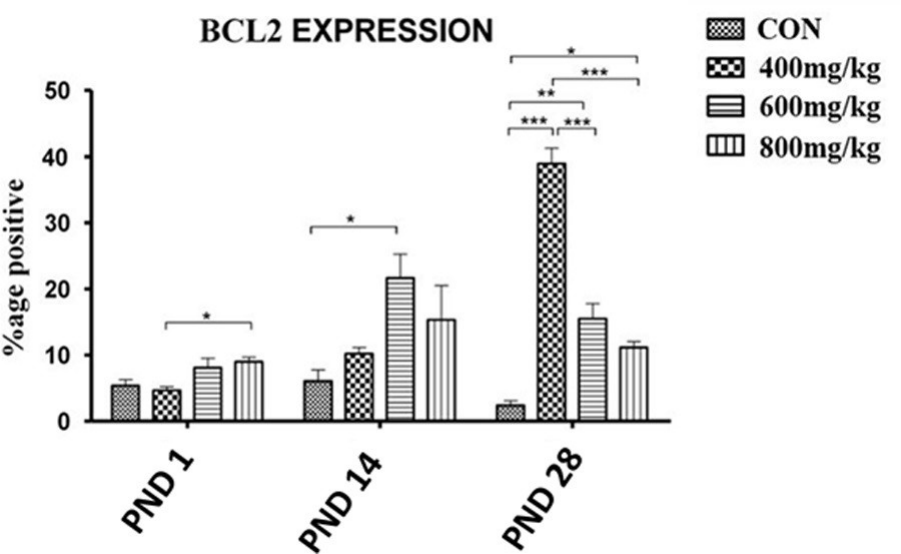
Dose-dependent Bcl-2 protein expression in both the control (CON) and kola nut-treated (400, 600, 800 mg/kg) groups (n = 5 pups). *P<0.05, **P<0.01 and ***P<0.001 for the indicated comparison.

There was a trend of dose-dependent increase in Bcl-2 protein expression in the kola nut-treated groups compared to the control. The expression in the TRT2 group quadrupled that of the control (P<0.05) on PND14. On PND 28, the expression in the TRT1 group was more than ten-fold (P<0.01) the control expression, whereas the expression in the TRT2 or TRT3 group was significantly less than that of TRT1, even though significantly higher than that of the control (Fig 15).

## Discussion

This study set out to investigate the potential effects of kola nut on the developing brain, focussing on the cerebellum as a region of the brain whose maturation is highly developmentally regulated.

The significantly lower body and brain weights observed up to PND 14 for pups from kola nut-treated dams as compared to control dams is in line with findings by Ikegwuonu et al. [18] and Umoren et al. [17], who reported a decrease in body weight when kola nut was administered to rats. Kola nut is well known to suppress the function of hunger centres in the brain and there is documentary evidence about ancient African soldiers who after taking kola nut could go on for days without food [16]. This known effect of kola nut could also explain the reduction in food intake observed in kolanut-administered dams days after administration of kola nut commenced, as well as the subsequent loss of weight that started weeks afterwards. The effects of kola nut are also known to be attributed to its constituents, for example theobromine and kolanin that aid the combustion of fats and carbohydrates and reduce the combustion of nitrogen and phosphorus in the body [34].

The organization of the Bergmann glial was observed to be disrupted by ingestion of kola nut, as demonstrated in the GFAP expression studies. The normal horizontal palisades parallel to the longitudinal plane of the folium were disrupted following the ingestion of kola nut. The Bergmann glial fibres extended from the cell bodies to the pial surface in the control group, just as described by Qu and Smith [35], but in the treated animals appeared short, irregular and scattered, consistent with the description by de Blas [36]. Bergmann fibres are a final stage in the development of a defined group of radial glia in the cerebellum. They help in guiding migrating immature neurones; extending through the intermediate zone to the pial region, they form the scaffold for Purkinje and other cells moving from the ventricular zone to their proper positions during embryogenesis [37]. Disorganization of the Bergmann glial cells, therefore, disrupted the migration of the young neurones [38], as has been observed in the delayed migration of the external granular cells [39–41]. It has been suggested that Bergmann glial fibres may be responsible for directing the geometrical organization of the cerebellar constituents [35].

As aforementioned, ingestion of kola nut during pregnancy in the rats caused aberrant, shortened and scattered Bergmann glial fibres, leading to granule cells’ migration defect in the cerebellum, with accumulation of cells in the external granular layer; this was also reported by Weller et al. [37]. While further research needs to be done to identify the mechanisms involved, Qu and Smith [35] reported that this disruption of the Bergmann glial fibres could be as a result of physical wrecking of glial guide scaffolding and sub-optimal neuronal glial guide communication during migration.

The Purkinje cells have five phases of maturation, with the first phase showing the dispersion and alignment into a monolayer with no or few synapses formed [42–45]. The kola nut-treated groups on PND28 showed Purkinje cell layering, which was abnormal. Kola nut treatment disrupted the normal maturation of the Purkinje cells, apart from the mentioned disruption of the Bergmann glial fibres. Purkinje cells are the only projection neurones of the cerebellum [42, 46]. Decrease in the Purkinje cell density, increased apoptosis, degenerated Purkinje cell axon, as well as granule cells with central chromatolysis were also observed in kola nut-treated groups. This is similar to the findings of Priyanka et al. [47] in a work involving aluminium-induced neurotoxicity in the cerebellum of albino mice. These were expected to reduce the cerebellar cortical outputs in the treated rats.

Kola nut treatment also induced status spongiosis, a term applied to the sieve-like appearance of nervous tissue associated with intracellular, extracellular, and intramyelinic accumulation of fluid and dissolution of myelin [48–51]. On PND 21 and 28, experimental groups that were exposed to kola nut extract developed spongiosum, which involved degeneration of the cerebellum alongside neuronal loss. This ‘cerebellar degeneration’ involved the noted destruction of axons with Purkinje cell loss as well as increased apoptosis of cerebellar granule cells. Liang et al. [49], who worked on neural-specific deletion of FIP200, a protein that has been associated with autophagy, found out that, upon deletion of this protein, there was degeneration of the cerebellum, with outcomes of neuronal loss, spongiosis, and neurite degeneration, similar to what we observed, as we also demonstrated the toxic effect on the cerebellum of certain doses of kola nut. While the mechanisms underlying these effects of kola nut have not been explored in these studies, a few possibilities have been suggested by some earlier studies, including vacuolation, mitochondrial dysfunction and generation of reactive oxygen species [51].

Furthermore, in kola nut-treated groups, cerebellar degeneration and astrogliosis were observed, as reflected in the over-expression of Bcl-2 and GFAP, respectively. Bcl-2 protein expression and glial activation could be induced by loss of neuronal cells [52, 53]. Astrocytes are reported by Sofroniew and Vinters [54] to be in ratio 5:1 to neurones. Astrocytes help in the formation of synapses by producing molecular signals and their loss or dysfunction can lead to demyelination [54]. They also help in regulating the blood vessel diameter and amount of blood flow. They react to any form of injury to the CNS, and this process is called reactive astrogliosis, which is a feature of many CNS pathological lesions. Astrocytes are supportive components of the neural tissue. However, reactive astrogliosis is a valid indicator of on-going pathology. It has also been established that astrocyte scars act as a neuroprotective barrier to inflammatory cells and they form border along damaged tissue [54]. The molecular marker of astrocytes, GFAP, was used in this study to detect the presence of reactive astrogliosis and cyto-architecture property. The upregulation of GFAP in the kola nut-treated groups with hypertrophy of individual astrocytes, as well as the overlap of processes with the neighbouring astrocytes and loss of individual domains, are features of injury to the CNS. Although the mechanism of the scar-formation was not explored in this study, it may have involved matured astrocytes that re-entered the cell cycle and parent cells in the local parenchyma or around the ventricle [55].

This study also showed increased expression of the anti-apoptotic protein Bcl-2 in kola nut-treated animals, although the effects of the various doses of kola nut were not consistently dose-dependent across the post-natal days examined. Alterations to Bcl-2 protein expression are used to determine the occurrence and intensity of programmed cell death (apoptosis) [50, 56]. The higher expressions of Bcl-2 seen with the administration of kola nut could indicate a reactive attempt by the cellular machinery to combat the ensuing damage by over-expressing Bcl-2, which is protective against apoptosis. While in the future we could additionally assess changes to the expression of pro-apoptotic members of the Bcl-2 family of proteins, such as Bax, and then determine the ratio of changes to Bcl-2 and Bax, the increase in Bcl-2 immuno-reactivity in this study suggests possible neuronal damage and these findings are corroborative of the findings of Baloui et al. [57].

In this study, the kola nut-treated rats had abnormally persistent external granular layer (EGL) up till day 21 after birth, with scanty viable cells in the molecular layer, thereby causing the molecular layer of the kola nut-treated groups to be thicker for age than in the control group and causing an increase in granule cell population. EGL has a high level of mitosis [58] and this increases the population of the granule cells. The thickness of EGL increases for approximately 10 days, then reduces, with the molecular layer replacing it. At postnatal day 21, it is expected for the EGL to have virtually disappeared, with molecular layer extending from the Purkinje cell layer to the pial surface [39, 58, 59]. This reduced radial migration of external granular cells through the molecular layer is a component of impaired cerebellar development [60, 61] resulting in hypercellular molecular layer [59], and this could cause abnormalities of movement and balancing in kola nut-treated rats. This observation warrants further study to ascertain the mechanisms by which kola nut alters the proliferation and persistence of the external granular layer.

This study raises certain health concerns that need to be addressed through wider studies, findings from which may necessitate public health campaigns to enlighten pregnant women in particular, and the entire population in general, about the potential risks of excessive or chronic consumption of kola nut. To establish some caveats, we acknowledge that this study was conducted using neonatal and juvenile rat brains as a means to understanding the potential effects of kola nut on the neonatal and juvenile human brains, and while there are similarities in the anatomy and biochemistry of the rat and human cerebella, there might also be subtle differences in their responses to various chemical agents. It is also possible that the doses examined in this study are not quite representative of, or might be higher than, equivalent doses in humans that pregnant women or other people who ingest kola nut are ever exposed to. Nevertheless, our work definitely shows that exposure to kola nut *in utero* or post-natally, at least at certain dose levels, whether through excessive acute consumption or chronic accumulative consumption, could be detrimental to brain health and function in neonates and young individuals. Even when such untoward effects do not translate to functional (phenotypic) deficits in affected individuals, it is possible that the exposure significantly increases their susceptibility to the detrimental effects of a number of pathological triggers that would otherwise not affect them significantly if they had not been so exposed to kola nut.

This study is a sensitisation towards further studies to examine the potential wider effects of kola nut on the developing brain. For example, how the fractions of the extract cross the blood brain barrier, including at what rate, should be looked into, and other abnormalities in the developing brain of pups from dams that had consumed kola nut during pregnancy should be checked. The underlying molecular mechanisms should also be investigated. To further embed the public health context and relevance of the work, surveys should be conducted to assess the pattern and rate of kola nut consumption amongst pregnant women and nursing mothers, which will, among other things, furnish information that could help to determine the equivalent range of doses that should be further examined in animal studies.

## Conclusion

This study shows that kola nut from *Cola nitida* has the capacity to disrupt normal development of rat cerebellum and induce neurotoxicity in the developing rat cerebellum, evidenced by delayed maturation and migration of neurones, astrocytic gliosis, apoptosis, demyelination of white matter and destruction of Bergmann glial and Purkinje cells. Further studies are needed to establish the wider implications of these observations for human brain development.
